# Visceral Adipose Tissue: A New Target Organ in Virus-Induced Type 1 Diabetes

**DOI:** 10.3389/fimmu.2021.702506

**Published:** 2021-08-04

**Authors:** Danny Zipris

**Affiliations:** Innate Biotechnologies LLC, Denver, CO, United States

**Keywords:** type 1 diabetes, Kilham rat virus, Inflammation, visceral adipose tissue (VAT), beta cells

## Abstract

Type 1 diabetes (T1D) is a proinflammatory pathology that leads to the specific destruction of insulin producing β-cells and hyperglycaemia. Much of the knowledge about type 1 diabetes (T1D) has focused on mechanisms of disease progression such as adaptive immune cells and the cytokines that control their function, whereas mechanisms linked with the initiation of the disease remain unknown. It has been hypothesized that in addition to genetics, environmental factors play a pivotal role in triggering β-cell autoimmunity. The BioBreeding Diabetes Resistant (BBDR) and LEW1.WR1 rats have been used to decipher the mechanisms that lead to virus-induced T1D. Both animals develop β-cell inflammation and hyperglycemia upon infection with the parvovirus Kilham Rat Virus (KRV). Our earlier *in vitro* and *in vivo* studies indicated that KRV-induced innate immune upregulation early in the disease course plays a causal role in triggering β-cell inflammation and destruction. Furthermore, we recently found for the first time that infection with KRV induces inflammation in visceral adipose tissue (VAT) detectable as early as day 1 post-infection prior to insulitis and hyperglycemia. The proinflammatory response in VAT is associated with macrophage recruitment, proinflammatory cytokine and chemokine upregulation, endoplasmic reticulum (ER) and oxidative stress responses, apoptosis, and downregulation of adipokines and molecules that mediate insulin signaling. Downregulation of inflammation suppresses VAT inflammation and T1D development. These observations are strikingly reminiscent of data from obesity and type 2 diabetes (T2D) in which VAT inflammation is believed to play a causal role in disease mechanisms. We propose that VAT inflammation and dysfunction may be linked with the mechanism of T1D progression.

## Introduction

Type 1 diabetes (T1D) is a multi-step proinflammatory pathology that culminates in the specific destruction of islet β-cells and lack of insulin secretion (1–3). The Centers for Disease Control and Prevention have estimated that ~1.25 million Americans are currently living with T1D and 40,000 new cases of T1D are being diagnosed in the U.S each year and it is estimated that five million Americans will live with T1D by mid-century (4).

It is thought that both genetic and environmental factors are key players in the mechanism that triggers diabetes (5–7). The risk for T1D development is substantially increased in relatives of T1D patients, since ~6% of children of a diabetic parent, 5% of siblings and 50% of monozygotic twins develop T1D compared to only 0.4% in the general population (8, 9). More than 50 T1D genetic risk loci have been identified to be associated with disease progression (10).

There is ample evidence from humans and animals supporting the notion that the environment plays a key role in mechanisms that trigger β-cell autoimmunity (11–18), and viruses have been postulated to play a pivotal role in these mechanisms (16, 17, 19–27). Due to ethical reasons, it is almost impossible to establish a causal role for microbial infections in triggering T1D, or address virus-induced disease mechanisms in humans. Furthermore, identifying microbes involved in triggering T1D may be hindered since by the time T1D is detected, the individual might have been infected with multiple viruses and the virus triggering the disease might have been cleared (28, 29). We have therefore used the BBDR and LEW1.WR1 rat models that develop T1D following infection with Kilham Rat Virus (KRV) (30) to identify how infections lead to β-cell inflammation and destruction.

Emerging evidence suggests that inflammation plays a key role in triggering numerous inflammatory disorders (31–35). We recently hypothesized that innate immune upregulation is associated with promoting virus-induced T1D (30, 36–43). Our recent data provided for the first time evidence linking inflammation in VAT with mechanisms of T1D (44). Inflammation in VAT is detectable soon after infection prior to insulitis and hyperglycemia and is characterized by infiltration of macrophage to the site of inflammation and proinflammatory cytokine and chemokine upregulation and tissue dysfunction (44). On the basis of these observations, we hypothesize that VAT inflammation and dysfunction may be associated with T1D mechanisms.

## Kilham Rat Virus

KRV is a rat-specific virus environmentally ubiquitous and a member of the *Parvoviridea*, a virus group of small single‐stranded DNA viruses with an average genome size of 5 Kbp encapsidated by protein in an icosahedral non‐enveloped particle (45). This virus group infects various animal species, including humans  (46) and rodents  (47). KRV encodes three overlapping structural proteins, VP1, VP2, and VP3, and two overlapping nonstructural proteins, NS1 and NS2  (47). There human parvovirus B19 has been linked with pro-inflammatory autoimmune disorders like acute myocarditis (48, 49), rheumatoid arthritis  (50), systemic lupus erythematosus (51), and Sjögren’s syndrome, as well as other autoimmune conditions  (50). Infection with B19 has been associated with the appearance of elevated levels of autoantibodies against nuclear antigens and double‐stranded DNA  (50). KRV infection can occur in natural environment leading to T1D without the need for virus injection (52). Known routes by which KRV transmission may occur are direct contact, aerosol, and oral (52).

## Rat Models of Virus-Induced T1D

There are two inbred rat strains that have been most used to address virus-induced T1D mechanisms, the BBDR and LEW1.WR1 rats. These animals are the only genetically un-manipulated animal models in which infection with a virus triggers anti-ß-cell autoimmunity (41). BBDR rats have normal levels and function of peripheral T cells (53, 54), and spontaneous diabetes does not develop in viral antibody–free BBDR rats (55). However, insulitis, hyperglycemia, and severe ketosis occur in animals after inducing innate immunity with Poly(I:C) plus elimination of regulatory ART2+ T cells (55), or following virus infection (52). T1D in the BBDR rat is mediated by the immune system since the transfer of lymph nodes from animals with diabetes to RT1u MHC compatible T cell deficient WAG nu/nu rats results in diabetes progression (56).

The LEW1.WR1 rat has also normal levels and function of T lymphocytes (57). The LEW1.WR1 rat has a higher degree of disease penetrance compared with that of BBDR rats as evidenced by the observation that elimination of ART2.1+ cells by itself can result in diabetes (57). As seen in the BBDR rat, KRV infection leads to hyperglycemia by specific loss of islet ß-cells, glycosuria, ketonuria, and polyuria (55, 57).

Infecting LEW1.WR1 and BBDR rats with KRV leads to specific β-cell inflammation, islet cell death and permanent T1D occurring following insulitis, 2-4 weeks following virus inoculation with disease rate of ~20 and 60%, respectively (30, 34, 35, 52). It is noteworthy that the ability of virus infection to trigger T1D or inflammation in the rat is not limited to KRV, since β-cell autoimmunity in the rat can be triggered by two other viruses, rat CMV (58). Furthermore, Poly I:C, a synthetic analogue of double stranded RNA which mimics viral infection, synergizes with low KRV titers, that by themselves do not induce T1D, on disease progression (41). Because double stranded RNA molecules can be expressed by different viruses, this may suggest that microbes other than KRV could also be associated with initiating T1D development (44). Indeed, multiple viruses have been hypothesized to be involved in triggering human T1D (5, 6, 16, 17, 19–26, 59).

A key factor linked with the mechanism leading to T1D in both animals and humans is likely to be linked with proinflammatory pathways that can potentially be upregulated by different virus groups (60). It is therefore plausible to hypothesize that while a human KRV homologue may not necessarily be involved in triggering T1D in humans, viruses that induce proinflammatory pathways similar to those induced by KRV may be linked with promoting β–cell autoimmunity in genetically susceptible individuals. Identifying mechanisms of KRV-induced T1D in rat models of virus-induced T1D could therefore provide valuable data on mechanisms mediating the human disease.

The relevance of the BBDR and LEW1.WR1 rat models to the human disease is supported by data from our laboratory and others. T1D in the rat better resembles the human disorder than the mouse model with respect to histopathology (61). Similar to the rat, there is no significant infiltration of immune cells around the islet (“peri-insulitis”) prior to disease onset and insulitis is morphologically mild and more similar to that detected in human T1D (62–64). As seen in humans, disease in the rat is not influenced by gender (65) and is MHC-dependent (61, 66).

The mechanism of T1D in the LEW1.WR1 rat is believed to be fundamentally different than that leading to T1D in the NOD mouse. In contrast to the rat, T1D development in the mouse is not dependent on microbial infections as germ-free mice retain the ability to develop disease (67). While *β-*cell autoimmunity in the mouse appears to be independent of the MyD88 signaling pathway (68), our studies demonstrated that the disease in the rat is mediated *via* the MyD88-TLR9 signaling axis (40). Finally, innate immune activation with exogenous activators of TLR2, TLR3/MDA-5, TLR4, TLR7/8, and TLR9, and exacerbates T1D in the rat (41, 69), but protects NOD mice from β–cell autoimmunity (70–73).

### Innate Immunity and Inflammation

Inflammation is a physiological reaction of the innate immune system to microbial infection or tissue injury leading to the secretion of numerous inflammatory mediators, such as cytokines and chemokines, which orchestrate cellular defense mechanisms and injured tissue repair (74, 75). In contrast to adaptive immunity that identifies antigenic molecules using highly specific receptors expressed on T and B lymphocytes, inflammation is the less specific arm of the immune system (76).

Innate immune sentinel cells such as dendritic cells (DCs), macrophages, and neutrophils and recognize invading microbes *via* pattern recognition receptors (PRRs) activating downstream innate immune pathways aiming to eliminate infections (77, 78). A key PRR group is the Toll‐like receptors (TLRs) family each member of which recognizes a different type of conserved pathogen-associated molecular patterns (PAMPs), such as TLR2 that senses cell wall molecules of gram-positive bacteria lipoteichoic acid and TLR3 and TLR9 that sense double stranded RNA and microbial DNA, respectively reviewed in refs. (79–88). Recognition of PAMPs by PRRs induces proinflammatory responses and activation of host defense mechanisms (79–88). The interaction of TLRs with their agonists induces in addition to proinflammatory cytokine and chemokine responses, the expression of MHC Class II and costimulatory molecules on antigen presenting cells (APCs), thus enabling these cells to effectively activate antigen-specific T cells to specifically attack invading pathogenic microbes (79–88). In addition to sentinel cells, innate immunity also has a humoral arm comprised of pattern recognition molecules (PRMs), such as lectin, ficolins, pentraxins, and the complement component C1q (89, 90).

### Role of Inflammation in KRV-Induced T1D

Infection with KRV induces a global innate immune upregulation detected in various lymphoid organs, such as the spleen, pancreatic lymph nodes, Peyer’s patches and thymus involving the induction of numerous proinflammatory cytokines, including IL-1ß, IFN-γ, and IL-12 3-5 days after infection, prior to insulitis and diabetes (30, 34, 35, 66). The rats develop humoral and cellular anti-KRV responses and clear the virus (91). We proposed that KRV-induced inflammation is associated with mechanisms of disease development (30, 36–43). We were the first to implicate TLR signaling in T1D progression (40, 41). We demonstrated that innate immune activation with ligands of TLRs synergizes with KRV infection on T1D development (41). Furthermore, we observed that the highly homologous H-1 parvovirus does not activate the innate immune system and fails to induce diabetes development in the BBDR rat (41). Our *in vivo* studies have shown that blocking IL-1 signaling with IL-1RA (39), or suppressing inflammation with a number of immunomodulatory agents, such as steroids (69), histone deacetylase inhibitor (38), antibiotics (30), or short chain fatty acids (92) prevents diabetes. Our hypothesis on the role of innate immunity in T1D is further supported by earlier data implicating TLR9 pathways in KRV-induced T1D mechanisms (40). We demonstrated that *in vitro* KRV-induced innate immunity is blocked by inhibitors of TLR9 and blockers of PKR and NF-κB (40). Finally, pharmacological suppression of TLR9 *in vivo* prevents T1D (40).

### COVID-19 and T1D

Given that COVID-19 induces robust inflammation in infected individuals, it has recently been hypothesized that this virus could potentially drive T1D *via* mechanisms associated in part with immune upregulation (93, 94). The data on the ability of COVID-19 to induce autoimmunity are mixed and clear evidence that COVID-19 activates anti-β–cell autoimmunity is not yet available (94–103). Moreover, the observations implicating COVID-19 in T1D development are based primarily on anecdotal data (95–101). Because hyperglycemia is only the end stage of the anti-islet autoimmune process that may start many years prior to disease onset (104–106), long-term follow up epidemiological studies will be required to determine whether COVID-19 infection increases the risk for T1D development in genetically-susceptible individuals.

## KRV-Induced Inflammation in Visceral Adipose Tissue (VAT)

In the course of our studies on the role of inflammation in virus-induced T1D, we observed that infection of LEW1.WR1 rats with KRV leads to inflammation in VAT detectable as early as day 1 post-infection, long before β-cell inflammation and hyperglycemia. This inflammation is characterized by an influx of CD68^+^ macrophages into VAT seen in the interstitial space surrounding adipocytes in KRV-infected animals but not control rats injected with PBS. In sharp contrast to VAT, subcutaneous adipose tissue (SAT) was observed to be free of cell infiltration (44). Activation of innate immunity with Poly (I:C) in the absence of virus also induces VAT inflammation. Because i.p. injection of KRV induces inflammation in proximal and distal organs, and since Poly (I:C) itself, in the absence of virus, can induce VAT inflammation, it is unlikely that the route of virus inoculation or site of infection play a critical role in triggering VAT inflammation. Unlike VAT, the exocrine tissue and islets from day 5-infected rats are insulitis-free, whereas ß-cells from day-14-infected animals are inflamed or show signs of tissue destruction (44). KRV induces the expression of virus transcripts and proinflammatory cytokines such as IL-1ß, IL-6, and IL-12p40 and chemokines in VAT *in vivo* and in purified adipocytes *in vitro* (44). Furthermore, KRV induces ER and oxidative stress response and activation of apoptotic pathways in infected VAT *in vivo* (44). KRV also downregulated the expression of adipokines and genes associated with mediating insulin signaling in VAT (44). Brief therapy with dexamethasone early in the disease course (days 1-5) prevents VAT inflammation and T1D. Based on these data, we hypothesized that VAT inflammation and dysfunction may be linked with early mechanisms of virus-induced disease development.

## Role of Adipose Tissue in Glucose Metabolism and Immunity

There are several types of adipose tissue, i.e. white adipose tissue (WAT), brown adipose tissue (BAT) and beige adipose tissue (107). WAT is the most abundant fat accounting for 5% to 50% of human body weight (107). It plays a key role in metabolic homeostasis by storing fat for long-term survival and by functioning as an endocrine organ (107–109). WAT is a main source of many adipokines, peptides or proteins with hormone-like properties that regulate metabolic homeostasis through local paracrine effects and endocrine effects (107–109). The metabolic characteristics of WAT is determined by its location in the body, commonly classified into subcutaneous fat and visceral fat depots (107–109). Adipose tissue can release and respond to cytokines and may therefore exert immune modulatory functions on non-adipose tissues (107). The discovery of leptin and adiponectin was the first indication that adipose tissue is an endocrine organ with the ability to regulate systemic energy homeostasis and glucose metabolism as well as mediate immunity. The metabolic effects of leptin and adiponectin on target tissues were observed to be robust (110).

Why KRV induces inflammation in VAT and not SAT is unclear. It may be that this is the result of differences in the function of VAT *versus* SAT. SAT is less active metabolically than VAT (111). It has been shown that adipocytes of VAT undergo more lipolysis than SAT and therefore contribute larger amounts of fatty acids to the circulation (111–113). On the other hand, SAT is considered to have a better capability of storing fatty acids, implying that it could store energy in periods of excess nutrition and supply fatty acids in periods of starvation (111).

Leptin has been suggested to play a key role in T2D development (reviewed in ref. 104). The long form of the leptin receptor (ObRb) capable of intracellular signaling is expressed in ß-cells, and exogenous leptin inhibits insulin production and secretion from human islets implying a direct action of leptin on β-cell function (105, 106, 114–118). Furthermore, mice deficient of leptin have increased appetite, weight gain, insulin resistance and diabetes, conditions that can be improved with leptin therapy (104, 119–124). In addition to its role in controlling energy balance, leptin can also influence immune functions reviewed in ref. (119). Indeed, macrophages express the leptin receptor (119) and leptin can increase the proliferation of monocytes and induce the expression of inflammatory cytokines such as TNF-α and IL-6 and other surface activation molecules (125).

Adiponectin has been shown to have beneficial effects on insulin sensitivity  (110, 126) and β-cell regeneration in mice with STZ-induced diabetes (127). Adiponectin has also been demonstrated to protect ß-cells from the detrimental effects of free fatty acids (128) *via* as yet unidentified mechanisms (118). Adiponectin is an endogenous insulin sensitizer in the skeletal muscle and liver, and administering mice with adiponectin results in lower blood glucose levels and the reversal of insulin resistance in mouse models of obesity (119). The receptor for adiponectin is expressed in macrophages, and adiponectin can suppress the production of TNF-α and IL-6 and induce the production of the anti-inflammatory mediators IL-10 and IL-1 receptor antagonist (119). Mice deficient in adiponectin have increased numbers of activated M1 macrophages in their adipose tissue with increased production of TNF-α, IL-6, and MCP-1 (119).

## Crosstalk Between Innate Immunity and Glucose Metabolism

The hypothesis that there is interplay between the innate immune system and glucose metabolism emerged after it was observed that administering low doses of lipopolysaccharide (LPS) leads to hyperglycemia mediated primarily by IL-1 pathways (129). Innate immune mediators such as IL-1 may play a beneficial role in maintain a normal glucose homeostasis by inducing insulin secretion and biosynthesis and β-cell proliferation reviewed in ref. (130).

In obesity, increased fat mass can result in adipocyte hypertrophy, hypoxia, death and ER stress response reviewed in refs. (119, 130). The adipose tissue death and dysfunction lead to the induction of chronic inflammation associated with the expression of proinflammatory cytokines such as IL-1, IL-6 and TNF-α and chemokines such as MCP-1 in adipocytes. MCP-1 and other chemokines released by adipocytes and immune cells in fat tissue further promote infiltration of monocytes and other immune cells into adipose tissue (130–132). Macrophages are the most abundant innate immune cells infiltrating and accumulating into adipose tissue of obese individuals (133).

Chronic inflammation in adipose tissue is believed to play a key role in the development of insulin resistance that is a hallmark of T2D in obese individuals reviewed in ref. (133). Insulin resistance may culminate in aberrant glucose uptake and glycogen synthesis (134). Consequently, ß-cells attempt to compensate for insulin resistance by increasing insulin secretion to restore normal glucose homeostasis (134). A further decline in insulin sensitivity makes the ß-cells exhausted, leading to hyperglycemia and T2D (135).

## VAT Inflammation and Dysfunction in KRV-Induced T1D *Versus* T2D

The underlying mechanisms and pathways critically involved in KRV-induced inflammation and T1D remain to be identified. The data from our laboratory implicating VAT inflammation and dysfunction in T1D development are highly reminiscent of observations from obesity and T2D in which VAT inflammation and dysfunction have been hypothesized to play a causal role in mechanisms that result in islet damage and diabetes progression (136–143). Although the level of the proinflammatory response detected in VAT from infected LEW1.WR1 rats is substantially greater than that typically seen in adipose tissue from T2D (44, 136, 137, 141, 142, 144–155), one cannot ignore the remarkable commonalities between inflammation observed in KRV-induced T1D *versus* T2D. Most notably, in both conditions, VAT is targeted by the innate immune system. Moreover, VAT inflammation in both disorders is linked with 1) macrophage infiltration into VAT, 2) expression of proinflammatory cytokines such as IL-1, IL-6 and TNF-α and as well as chemokines such as CCL2, CCL5, and CXCL-10, 3) oxidative stress response, 4) apoptosis, 5) adipocyte death, and 6) tissue dysfunction (136, 137, 141, 142, 144–155).

## Role of VAT Inflammation and Dysfunction in Virus-Induced T1D

**Figure 1:**
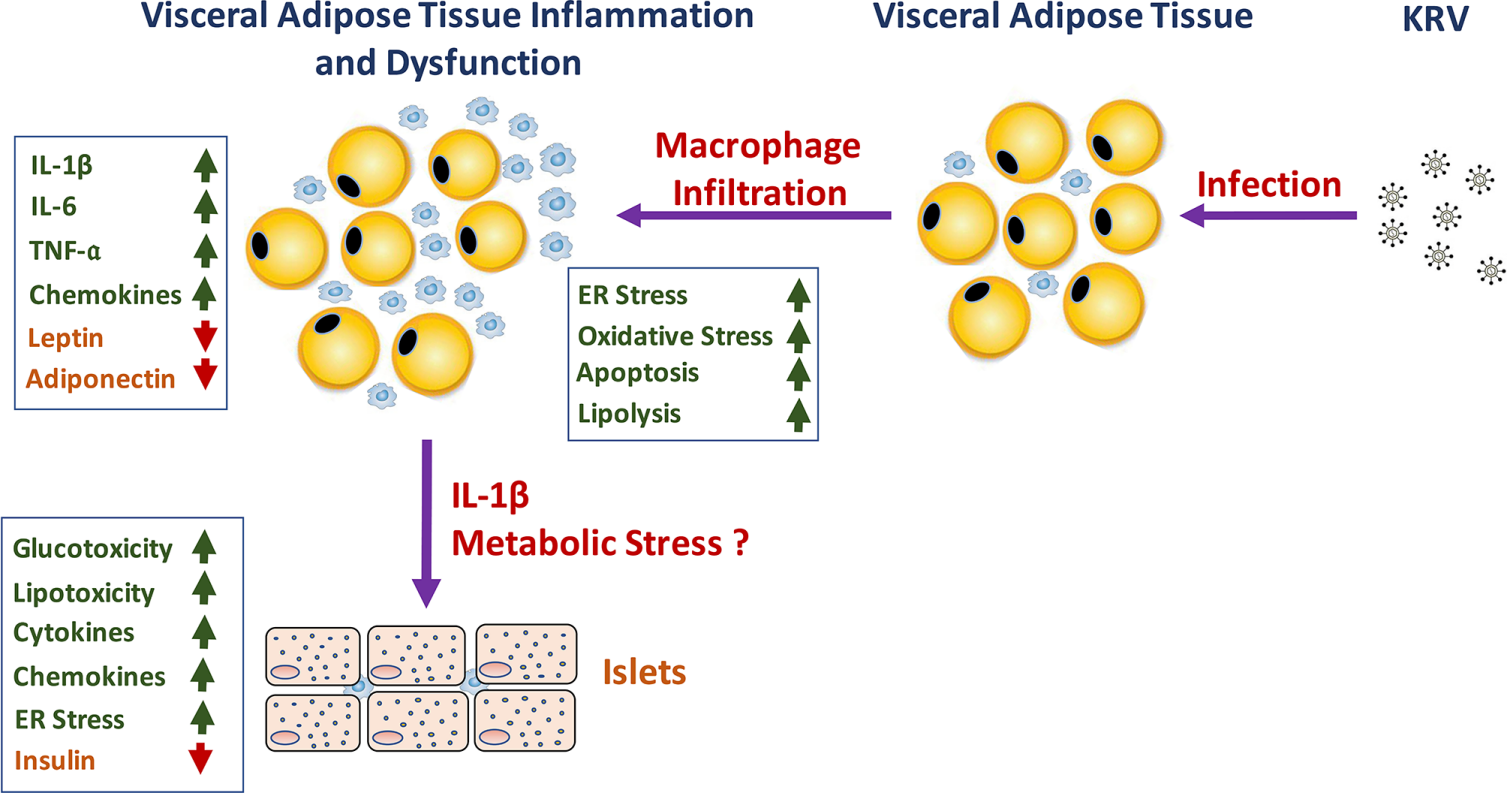
A schematic representation of the hypothesized role of KRV-induced VAT inflammation and dysfunction in the mechanism of KRV-induced T1D.

Whether and how VAT inflammation and dysfunction play a role in KRV-induced T1D mechanisms remain to be further elucidated. We propose a model that may explain how VAT inflammation and dysfunction lead to T1D (see model in **Figure 1**). We hypothesize that infection with KRV results in VAT infection and TLR-induced macrophage activation and infiltration into VAT. Inflammation in VAT associated with a robust proinflammatory cytokine response may lead to adipose tissue hypoxia, ER and oxidative stress responses and apoptosis and consequently aberrant adipokine expression (118, 119, 133, 156, 157). In Obesity, free fatty acids, and lipid intermediates synergistically induce adverse effects on both β-cell mass and function contributing to the progressive loss of functional β-cell mass reviewed in ref. (118). Likewise, circulating factors such as cytokines released from inflamed tissues such as adipose tissue and activated innate immune cells can adversely affect ß-cells by impairing their functions and limiting cell mass (118). In a similar manner, KRV-induced excessive lipolysis resulting from adipocyte death can result in excess of free fatty acids in the circulation, which can induce lipotoxicity. KRV-induced proinflammatory cytokines such as IL-1ß can enter the circulation and from there to the pancreas where it may exert toxic effects on islets, potentially leading to metabolic and cellular stress in ß-cells (136, 158). Furthermore, a rise in glucose levels in the microenvironment of ß-cells can activate the inflammasome in pancreatic ß-cells, further increasing the expression of IL-1ß (136, 158). Consequently, IL-1ß released from ß-cells may trigger the recruitment and activation of innate immune cells, which may then release more IL-1ß. IL-1ß in the islet microenvironment can exacerbate ß-cell dysfunction, and trigger apoptosis in ß-cells (30, 36, 40, 136, 158). Finally, islet impairment and damage may ultimately signal innate and adaptive immunity to attack and destroy ß–cells leading to permanent hyperglycemia (159–162).

## Conclusions and Future Perspectives

Earlier data demonstrated that the mechanism of KRV-induced T1D is associated with innate immune activation early in the disease course. We recently reported that infection with KRV results in VAT inflammation and dysfunction detected soon after infection. There are marked similarities between inflammation detected in VAT from infected LEW1.WR1 rats and inflammation detected in VAT from T2D patients. Whether as found in T2D, a cause-and-effect relationship exists between VAT inflammation and islet autoimmunity remains to be determined. As discussed in this Review, there is crosstalk between the innate immune system and glucose metabolism. We propose a paradigm by which virus-induced global innate immunity resulting in proinflammatory cytokine and chemokine upregulation and aberrant adipokine profile and lipolysis in VAT lead to metabolic stress and β–cells inflammation and destruction. Future studies will identify the interplay between the innate immune system and metabolic pathways and its role in triggering virus-induced disease. Identification of critical metabolic and immune pathways linked with β–cell autoimmunity will open new avenues for the development of targeted therapies for disease amelioration.

## Author Contributions

The author confirms being the sole contributor of this work and has approved it for publication.

## Conflict of Interest

DZ was employed by Innate Biotechnologies, LLC.

## Publisher’s Note

All claims expressed in this article are solely those of the authors and do not necessarily represent those of their affiliated organizations, or those of the publisher, the editors and the reviewers. Any product that may be evaluated in this article, or claim that may be made by its manufacturer, is not guaranteed or endorsed by the publisher.
